# Anti-Oxidant, Anti-Mutagenic Activity and Safety Evaluation of Antrocin

**DOI:** 10.3390/toxics11060547

**Published:** 2023-06-20

**Authors:** Yi-Hui Su, Jia-Shuan Wu, Yan-Zhen Dai, Yng-Tay Chen, Yan-Xiu Lin, Yew-Min Tzeng, Jiunn-Wang Liao

**Affiliations:** 1 Graduate Institute of Veterinary Pathobiology, National Chung Hsing University, Taichung 402, Taiwan; 2 Graduate Institute of Food Safety, National Chung Hsing University, Taichung 402, Taiwan; 3 Research Center for Animal Medicine, National Chung Hsing University, Taichung 402, Taiwan; 4 Department of Applied Science, National Taitung University, Taitung 950, Taiwan

**Keywords:** antrocin, anti-oxidant capacity, genotoxicity, 28-day oral toxicity study

## Abstract

Antrocin is a novel compound isolated from *Antrodia cinnamomea*, and is classified as a sesquiterpene lactone. The therapeutic efficacy of antrocin has been studied, and it has shown an antiproliferative effect on various cancers. The aim of this study was to evaluate the anti-oxidant activity, potential genotoxicity, and oral toxicity of antrocin. Ames tests with five different strains of *Salmonella typhimurium*, chromosomal aberration tests in CHO-K1 cells, and micronucleus tests in ICR mice were conducted. The results of anti-oxidant capacity assays showed that antrocin has great anti-oxidant activity and is a moderately strong antimutagenic agent. In the results of the genotoxicity assays, antrocin did not show any mutagenic potential. In the 28-day oral toxicity test, Sprague Dawley rats were gavaged with 7.5 or 37.5 mg/kg of antrocin for 28 consecutive days. In addition, 7.5 mg/kg sorafenib, an anti-cancer drug, was used as a positive control for toxicity comparison. At the end of the study, antrocin did not produce any toxic effects according to hematology, serum chemistry, urine analysis, or histopathological examinations. According to the results of the genotoxicity and 28-day oral toxicity study, antrocin, at a dose of 37.5 mg/kg, did not cause adverse effects and can be a reference dose for therapeutic agents in humans.

## 1. Introduction

Reactive oxygen species are natural by-products in the process of cell energy production. Oxygen-centered reactive oxygen radicals include superoxide anion, hydrogen peroxide, and hydroxide free radicals, which play an important role in cell signaling, energy metabolism, and redox balance in the body [1]. The increase in external environmental pressures, such as ultraviolet radiation, air pollution, ionizing radiation, and smoking, will cause the concentration of reactive oxygen species to increase rapidly, resulting in the inability of organisms to neutralize excessive reactive oxygen species. Excessive free radicals in the body can cause cell damage and oxidative stress, alter DNA sequences, cause genetic mutations, lead to malignant mutations in cells, and produce tumors, in addition to damaging other cellular components such as proteins, enzymes, and cell membranes [2]. Therefore, many studies have been devoted to finding natural substances with high antioxidant properties that are safe and non-toxic.

Traditional Chinese medicine has been used for more than a thousand years in Asia. The majority of Chinese medicine consists of plants or fungi. Although it is not fully understood how they work, medicinal herbs present some surprising functions in healing [3,4]. *Antrodia cinnamomea* (AC) is a unique endemic fungus from Taiwan. It has been used as an edible herb for indigenous people in Taiwan for many years. Since the 1990s, more and more scientists have invested in the research of AC and its ingredients. More than one hundred different compounds have been isolated from AC, and the percentage of each compound may vary depending on different culture methods [5,6]. Most of the compounds belong to polysaccharides, benzenoids, diterpenes, triterpenoids, steroids, and maleic and succinic acid derivatives. Although AC has been shown to have different medicinal effects, the mechanisms are still not fully understood. In retrospective studies, AC has been used for liver protection, immunomodulation, as an antioxidant, and as an anticancer drug. Among these effects, the potential for being a therapeutic anticancer drug has grabbed much attention [7,8]. Since cancer causes many deaths all over the world and remains a major health concern, finding ways to fight cancer is still a big challenge.

Antrocin, a sesquiterpene lactone first isolated from AC in 1995, has been reported for its antiproliferative properties against various cancers in vitro and in vivo [9]. Not only can antrocin be extracted from the fruiting bodies of AC, but a few synthetic methods have also been mentioned, thus widening the applications of antrocin [10,11]. Antrocin has shown anti-tumor effects in breast cancer, lung cancer, and bladder cancer by inhibiting different tumor cell proliferation signal pathways [12,13,14]. In vivo intraperitoneal administration of antrocin (5 mg/kg/day) significantly suppressed the growth of lung cancer tumor xenografts in non-obese diabetic/severe combined immunodeficiency mice, indicating that antrocin may be a potential therapeutic agent for human lung cancer cells through constitutive inhibition of the JAK2/STAT3 pathway [14]. It also enhanced the sensitivity of radioresistant prostate cancer when combined with radiotherapy [15]. However, despite its therapeutic effects, the toxicity of antrocin remains unknown. In our study, we aimed to evaluate its anti-oxidant and antimutagenic activities in vitro, along with its safety, such as genotoxic effects, by using a bacterial reverse mutation test (Ames test), an in vitro mammalian chromosomal aberration test, and an in vivo mammalian erythrocyte micronucleus test. Furthermore, a 28-day oral toxicity test of antrocin in Sprague Dawley (SD) rats with sorafenib as a positive control was conducted to ensure the safety of antrocin.

## 2. Materials and Methods

### 2.1. Test Material

**Figure 1:**
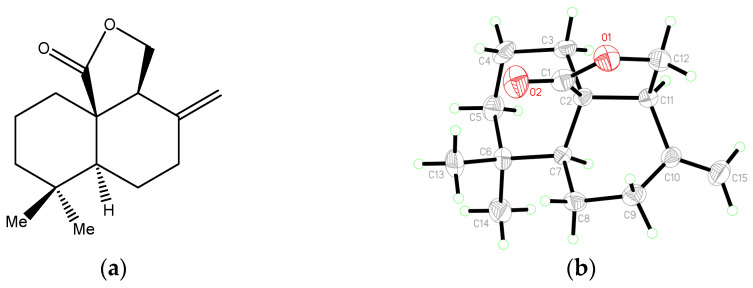
The chemical structure of (-)-Antrocin, (**a**): chemical formula of antrocin (**b**): the Oak Ridge Thermal Ellipsoid Plot (ORTEP) diagram of (-)-Antrocin.

Optical pure antrocin, with a purity > 95%, was provided by Professor Zhen Yang at the School of Chemical Biology and Biotechnology, Peking University. The chemical formula of antrocin is showed in Figure 1 and Table 1. Antrocin was dissolved in 95% ethanol, and the solubility was about 133 mg/mL. A 50 mg/mL ethanol solution of antrocin was prepared as a stock solution, and was diluted before testing. All of the samples were stored at 4 °C.

### 2.2. Safety Evaluations

#### 2.2.1. Ames Test

The Ames test was conducted to evaluate the mutagenic potential of antrocin using a plate incorporation test with or without S9 metabolic activation [16,17]. Histidine-dependent *Salmonella typhimurium* strains TA98, TA100, TA102, TA 1535, and TA1537 were used, and the genotypes of the bacterial strains were confirmed using *uvrB* mutation, lipopolysaccharide defect, histidine mutation, and ampicillin resistance. A bacterial toxicity test was performed in five different strains of *Salmonella typhimurium* prior to the assay to detect the cytotoxic level of antrocin, and the results showed that the maximum non-toxic concentration of antrocin was 0.05 mg/plate in all tester strains. Thus, the following concentrations of antrocin were tested in the Ames test: 0.003125, 0.00625, 0.0125, 0.025, and 0.05 mg/plate. We used 100% ethanol as a negative control, and chemicals were used as positive controls. Briefly, 100 μL antrocin (0.003125, 0.00625, 0.0125, 0.025, and 0.05 mg/plate), 100 μL 16–18 h cultured *Salmonella* tester strains (approximately 1 × 10^9^ cells/mL), 200 μL 0.5 mM Histidine/biotin, and 200 μL S9 mixture (when being tested), were mixed in soft agar. After slight vortexing, the solution was poured onto an MA plate, and incubated at 37 ± 1 °C for 48–72 h. All of the colonies were counted. It was considered potentially mutagenic if the sample caused a dose-dependent increase in the mean number of revertant colonies, at least a double increase, compared to the negative control.

#### 2.2.2. In Vitro Chromosomal Aberration Test

A chromosomal aberration test was performed to evaluate the potential for antrocin to induce structural chromosomal abnormalities in Chinese hamster ovary cells (CHO-K1 cells). The test followed the protocol in *Genetic Toxicology Testing: A Laboratory Manual* [18]. CHO-K1 cells were obtained from the Bioresource Collection and Research Center (BCRC), and were cultured in Ham F-12 medium with 10% fetal bovine serum and 0.1% Penicillin–Streptomycin at 5% CO_2_ in a 37 °C incubator. An MTT assay was conducted to test for cytotoxicity, and the half maximal inhibitory concentration (IC_50_) of antrocin was about 122.7 μg/mL. Hence, the dosages of 25, 50, and 100 μg/mL of antrocin were selected for the chromosomal aberration test. Three experimental conditions were conducted: 3 h with and without S9 (short term), and 19 h without S9 (long term). Fresh culture medium served as a negative control, and 250 µg/mL cyclophosphamide (with S9) and 25 µg/mL mitomycin C (without S9) were used as a positive control. CHO-K1 cells were cultured in 25T flasks, incubated with samples for 3 or 19 h with and without S9, and harvested at the time of 1.5 cell cycle lengths (about 21 h). Two hours before harvesting, 100 μL colcemid (10 μg/mL) was added to disrupt cell mitosis. Collected cells were then treated with 0.6% KCl solution and fixed with acetic acid–methanol (1:3) solution. After fixation, cells were stained with Diff-Quik on a glass slide. A total of 300 cells in metaphase per group were observed, and the number of chromosomal aberrations was recorded. The chromosomal aberration rate (%) was calculated using the following formula: (number of cells with aberrations/total number of cells examined) × 100. A Student’s *t*-test was used for statistical analysis, and a significant increase (*p* < 0.05) compared to the negative control indicated a positive response.

#### 2.2.3. In Vivo Mammalian Erythrocyte Micronucleus Test

The study followed the Organization for Economic Cooperation and Development (OECD) test guideline No. 474 with some modifications, and was approved by the Institutional Animal Care and Use Committee (IACUC) of National Chung-Hsing University (IACUC: 107-131) [19]. Twenty-five ICR male mice were purchased from BioLASCO Taiwan Company Ltd. (Taipei, Taiwan). The animal housing facility was maintained at 21 ± 2 °C with 50–65% humidity under a 12 h light/dark cycle. Laboratory Autoclavable Rodent Diet 5010 (LabDiet, St. Louis, MO, USA) and water ad libitum were supplied. Mice were grouped as either negative control (olive oil with 5% ethanol), three dosages of antrocin (100, 125, and 250 mg/kg), and positive control (cyclophosphamide 60 mg/kg). Except for cyclophosphamide, which was administered via intraperitoneal injection, the negative control and antrocin groups were given by oral gavage. Mice were weighed and monitored for clinical signs daily for three days after the treatment. At 48 and 72 h post-treatment, peripheral blood samples (about 1–2 μL) were collected from the retro-orbital region under isoflurane anesthesia. All of the samples were prepared according to the MicroFlow PLUS-Mouse kit instruction manual (version 170503) for flow cytometric analysis. A flow cytometer BD-FACScan (Becton Dickinson, Franklin Lakes, NJ, USA) with 488 nm excitation was set up for the study. A total of 200,000 erythrocytes were scored for the percentage of polychromatic erythrocytes (PCE). The percentage of reticulocytes and reticulocytes with micronuclei was calculated.

#### 2.2.4. 28-Day Oral Toxicity Test

This study followed the OECD Guideline for Testing of Chemicals: Repeated Dose 28-Day Oral Toxicity in Rodents No. 407, with some modifications [20], and was approved by the IACUC of National Chung-Hsing University (IACUC: 106-098). In this study, 5-week-old Sprague Dawley rats from BioLASCO Taiwan Company Ltd. (Taipei, Taiwan) were used. The animal housing conditions were as mentioned previously. All of the rats received Laboratory Rodent Diet 5001 (LabDiet, St. Louis, MO, USA) and water ad libitum. Rats were randomly separated into three groups (5/sex/group) consisting of a negative control (olive oil), positive control (sorafenib 7.5 mg/kg), antrocin 7.5 mg/kg, and 37.5 mg/kg. It is known that in vivo intraperitoneal administration of antrocin (5 mg/kg/day) significantly suppresses the growth of lung cancer tumor xenografts in mice [14]. Due to difficult synthesis of antrocin, the sample production is limited; the toxicology dosages were selected based on the therapeutic dosage (5 mg/kg/day), 7.5 and 37.5 mg/kg/day (approximately 1.5 and 7.5 times the therapeutic dosage, respectively) in this study. Samples were prepared fresh daily before the treatment. A total of 30 rats were used in the test. Rats were orally administered 10 mL/kg body weight of the samples once a day for 28 consecutive days. During the period of the study, clinical signs and mortality were recorded daily, and feed consumption and body weights were recorded weekly. After 28 days of treatment, all the rats were sacrificed. Blood samples were collected for hematology (Sysmex XE2100, Kobe, Japan), clinical chemistry (ADVIA 1800, Siemens, NY, USA), and urinalysis (Clinitex 100 Urine Chemistry Analyzer, Miles Inc. Diagnostic Division Ellchart, IN, USA). Necropsy and gross examinations were performed, and organs, including the brain, heart, thymus, liver, spleen, kidneys, adrenal glands, testes (males), and ovaries (females) were weighed. For pathological analysis, all organs of rats in the control and high-dose groups underwent histopathological examination. All gross lesions, brain (representative regions including cerebrum, cerebellum, and medulla/pons), spinal cord (at three levels: cervical, mid-thoracic, and lumbar), pituitary, thyroid, parathyroid, thymus, esophagus, salivary glands, stomach, small and large intestines, liver, pancreas, kidneys, adrenals, spleen, heart, trachea and lungs, aorta, ovaries, uterus, cervix, vagina, testes, epididymides, prostate, seminal vesicles, coagulation glands, mammary gland (female), urinary bladder, lymph nodes, peripheral nerve, skeletal muscle, bone, bone marrow, skin, and eyes were fixed in 10% neutral buffered formalin, trimmed, embedded, and H&E stained for histopathological examination. The semi-quantitative criteria of histopathological examination were evaluated by a veterinary pathologist with certification in Taiwan (CSVP Vet Path No. 0019). The incidence and semi-quantitative score system are recommended by Shackelford et al. [21]. The degree of lesions in each item is graded from one to five depending on severity: 1 = minimal (<1%); 2 = slight (1–25%); 3 = moderate (26–50%); 4 = moderate/severe (51–75%); and 5 = severe/high (76–100%).

### 2.3. Anti-Oxidant Activity

#### 2.3.1. Total Polyphenol Content

We added 40–50 μL of sample to a 96-well plate and made it up to 100 μL with ddH_2_O. For each sample, a parallel sample was prepared as a sample background control. Vanillic acid was used as a positive control. We added 50 μL of 50 mM vanillic acid solution per well to the desired wells and made it up to 100 μL with ddH_2_O. We adjusted the volume of all standard curves to 100 µL with ddH_2_O. We then added 20 μL PC (Phenolic Compounds) Probe to the standard curve and sample reaction wells, and 20 μL PC Assay Buffer to the sample background wells. For standard curve preparation, we added 0, 2, 4, 6, 8, and 10 μL of 1 mM catechin standards to clear 96-well plates to generate 0, 2, 4, 6, 8, and 10 nmol/well catechin standard, respectively. We adjusted the volume of all standard curves to 100 µL with ddH_2_O. We added 80 μL of PC Assay Buffer to the standard curve, samples, and sample background wells. We incubated the 96-well plate for 10 min at room temperature (24–26 °C) with gentle rocking. The absorbance was measured at 480 nm.

We subtracted the 0 nmol standard OD480 value from all standard curve readings and drew a catechin standard curve. If the sample background well reads were significant, the sample background control reads were subtracted from their paired sample reads.

The background-corrected sample OD480 values were applied to the catechin standard curve to obtain the B nmol product (diazo chromophore) produced during the reaction.

The following calculation was used to determine the mM catechin equivalents of the sample:

Sample phenolic compound concentration (mM catechin equivalents)

B/V × D = nmol/µL (1)

B stands for the amount of diazo chromophore, calculated from the standard curve (nmol of catechin); D stands for the sample dilution factor (for undiluted samples, D = 1); V stands for the volume of sample added to the well (in µL).

#### 2.3.2. Ferric Reducing Ability of Plasma (FRAP)

The following methodology describes the FRAP assay: Add 10 µL of sample to a 96-well plate. For each sample, a parallel sample was prepared as a sample background control. Add 4 µL of FRAP Positive Control to each well and make up to 10 µL with FRAP Assay Buffer. For standard curve preparation, add 0, 2, 4, 6, 8, and 10 µL of a 2 mM ferrous standard solution to the wells of a 96-well plate to generate 0, 4, 8, 12, 16, and 20 nmol/well of ferrous iron, respectively. Adjust the volume to 10 µL/well with FRAP Assay Buffer. Mix sufficient reagents for the number of tests to be performed, including: Add 190 µL of Reaction Mix to the wells of the standard curve, positive control, and test samples. Add 190 µL Background Control Mix to the sample background control wells and mix well. After 60 min of reaction at 37 °C, measure the absorbance at 594 nm. Subtract 0 nmol standard readings from all standard curve readings to draw a ferrous standard curve. If the sample background control is significant, subtract the background control reading from its paired sample reading. Apply the sample OD value to the ferrous standard curve to obtain the reduced ferrous ion B nmol generated during the reaction.

Use the following calculation to determine the mM ferrous equivalence of the sample.

Sample FRAP or mM ferrous equivalents =

B × D/V = nmol/µL(2)

B = amount of ferrous ammonium sulfate from standard curve (nmol)
D = dilution factor
V = volume of sample added to the well (µL)

#### 2.3.3. Trolox Equivalent Antioxidant Capacity (TEAC)

The following methodology describes the TEAC assay: Add 40 μL of sample to a 96-well plate and adjust the volume to 100 μL with ddH_2_O. The absorbance of the sample should be within the linear range of the standard curve (0–20 nmol/well). The detection limit of this assay is approximately 0.1 nmol (or 1 µM) Trolox per well. For Trolox Standard Curve Preparation, dissolve lyophilized Trolox Standard in 20 µL of pure DMSO, add 980 µL of distilled water and mix well to generate a 1 mM solution, then aliquot the solution and store it at −20 °C for stable storage for 4 months. Add 0, 4, 8, 12, 16, 20 µL of Trolox standard to each well. Adjust the total volume to 100 µL with ddH_2_O to obtain 0, 4, 8, 12, 16, 20 nmol of Trolox standards, respectively. For preparation of working solutions, dilute 1 part of Cu^2+^ reagent with 49 parts of Assay Diluent, and dilute enough working solution for detection. Add 100 µL of Cu^2+^ working solution to standard wells and sample wells. After capping the plate and reacting at room temperature for 1.5 h, measure the absorbance at 570 nm. Draw a Trolox standard curve by subtracting the 0 nmol standard reading from all standard curve readings. Sample antioxidant Trolox equivalent concentration:

Sample antioxidant capacity = *Sa*/*Sv* = nmol/µL or mM Trolox equivalent (3)

*Sa* = the amount of sample (in nmol) read from the standard curve.
*Sv* = the undiluted sample volume added to the well.

### 2.4. Antimutagenicity Test

Based on bacterial toxicity tests of Ames test, the highest non-toxic concentration of 0.05 mg/plate in *Salmonella typhimurium* TA98 and TA1535 was determined. For the antimutagenicity test, the concentrations of antrocin were 0.05, 0.025, and 0.0125 mg/plate with or without S9 fraction in *Salmonella typhimurium* TA98 and TA1535. The negative control group contained sterile ethanol. The methodology was as follows: Add 100 μL of the non-toxic concentration antrocin solution and 200 μL 0.5 mM Histidine/Biotin solution, 100 μL of cultured bacterial solution, respectively. For the group treated with liver activating enzyme, add 200 μL S9 mixture (for cultures with metabolic activation) to 2 mL of soft agar at 45 °C and mix evenly. After mixing, pour it onto the minimal glucose agar plates (MA). Shake gently until the soft agar is evenly spread on the agar plate. After coagulation at room temperature, incubate the Petri dishes upside down at 37 °C for 48 h, and then count all colonies in each plate. The number of His^+^ revertant colonies was counted, and each test concentration needed to be repeated three times. Inhibition of sample antimutagenicity [22]:

(4) $$\mathrm{Inhibition}\left(\%\right)=\left(1-\frac{a-c}{b-c}\;\right)\times 100$$

a = number of revertant colonies in presence of sample
b = number of revertant colonies of positive control (without sample)
c = spontaneous revertants

If the inhibition is less than 25%, it is a weak antimutagenic agent; if the inhibition is between 25% and 40%, it is a moderately strong antimutagenic agent; if the inhibition rate is higher than 40%, it is a strong antimutagenic agent [23].

### 2.5. Statistical Analysis

The data was expressed as mean ± standard deviation, and the difference between the control and treatment groups was determined by the Student’s *t*-test. All the statistical analyses were conducted using MicroSoft Excel software and the statistical significance levels were determined by a 2-tailed test (*p* < 0.05)

## 3. Results

### 3.1. Safety Evaluations

#### 3.1.1. Ames Test

The number of revertant colonies is summarized in Table 2. The number of revertant colonies in the negative control groups and all the antrocin groups were within the historical range in our records. There were no significant changes in the number of colonies for antrocin on five *Salmonella typhimurium* tester strains with or without metabolic activation. In the positive control groups, there was a more than two-fold increase in the number of revertant colonies. Taken together, no significant effects of antrocin were evident in five *Salmonella typhimurium* tester strains, suggesting that antrocin had no mutagenic effect on TA98, TA100, TA102, TA 1535, or TA1537.

#### 3.1.2. In Vitro Chromosomal Aberration Test

In the MTT assay, after incubation with CHO-K1 cells for 24 h, the cell viabilities of antrocin at the concentrations of 31.25, 62.5, 125, 250, and 500 μg/mL were 81.3 ± 5.0%, 62.9 ± 8.9%, 30.0 ± 1.2%, 24.8 ± 1.9%, and 22.1 ± 1.5%, respectively. The IC_50_ of antrocin in CHO-K1 cells was about 122.7 μg/mL. Based on the results of the MTT assay, antrocin at the concentrations of 25, 50, and 100 μg/mL was used in the chromosomal aberration test.

The results for the chromosomal aberration assay are shown in Table 3. In the 3 h, S9-absent group, the aberration frequencies at 25, 50, and 100 μg/mL were 4.3 ± 3.8%, 4.3 ± 2.9%, and 5.3 ± 2.1%, respectively. In the 3 h S9-present group, the aberration frequencies at 25, 50, and 100 μg/mL were 5.3 ± 1.2%, 6.3 ± 2.1%, and 6.3 ± 2.1%, respectively. In the 19 h S9-absent group, the aberration frequencies at 25, 50, and 100 μg/mL were 9.3 ± 1.2%, 10.3 ± 2.5%, and 7.3 ± 1.5%, respectively. In the positive control groups, the chromosomal aberration frequencies were 14.7 ± 2.3%, 15.3 ± 2.3%, and 32.0 ± 6.0% in the 3 h S9-absent, 3 h S9-present, and 19 h S9-absent groups, respectively, which was significantly increased (*p <* 0.05) compared to the negative control groups. In summary, on short-term exposure with S9, short-term exposure without S9, and long-term exposure groups, there were no significant differences in the chromosomal aberration frequency in CHO-K1 cells between the negative control and antrocin groups. In regard to mammalian cells, antrocin showed cytotoxicity not only in different kinds of tumor cells at varying doses, but also in CHO-K1 cells. However, no potential genotoxic effects of antrocin were found. Further tumor specificity and cytotoxicity tests need to be carried out.

#### 3.1.3. In Vivo Mammalian Erythrocyte Micronucleus Test

No clinical signs or mortalities were observed during the test period, and there were no significant differences in the body weight change between the test groups. The results of the micronucleus test are shown in Table 4. The frequencies of micronuclei after treatment with antrocin at 100, 125, and 250 mg/kg were 20.4 ± 2.4‰, 20.8 ± 2.5‰, and 18.8 ± 1.9‰ at 48 h, and 31.0 ± 6.1‰, 28.9 ± 5.0‰, and 26.5 ± 4.6‰ at 72 h, respectively. The frequencies of micronuclei at 48 and 72 h were 3.6 ± 2.1‰ and 3.9 ± 2.1‰ in the positive control group, which was significantly decreased compared to the control group, indicating chromosomal damage caused by cyclophosphamide. Based on the results, there were no significant changes in the frequency of micronuclei in the antrocin-treated groups after 48 or 72 h.

#### 3.1.4. 28-Day Oral Toxicity Test

**Figure 2:**
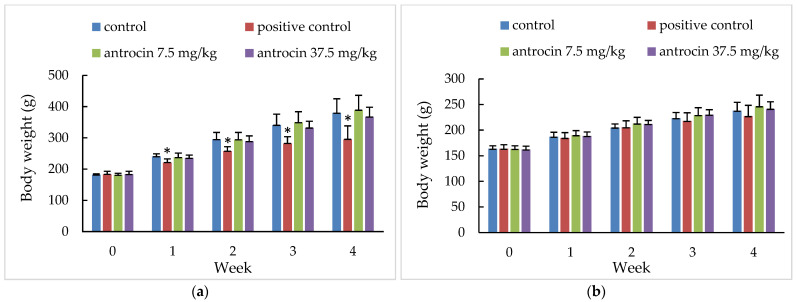
Body weight changes of rats treated with antrocin in the 28-day oral toxicity study. Body weight changes are listed in male (**a**) and female (**b**) rats. Control: Olive oil, positive control: sorafenib 7.5 mg/kg, antrocin 7.5 mg/kg bw, and antrocin 37.5 mg/kg bw. * Significant difference between the control and treated groups at *p* < 0.05.

No deaths or treatment-related abnormalities occurred in the control or antrocin-treated groups during the test period. In the positive control group, one male rat presented with rough hair and depression after day 14. Body weight change is shown in Figure 2. For mean body weight (g, %) and feed consumption (g), there were no significant changes in the antrocin-treated groups compared to the control group. In the positive control group, sorafenib 7.5 mg/kg, mean body weight (g), and feed consumption (g) in male rats were significantly decreased (*p <* 0.05) at the intervals of week 1, 2, 3, and 4 compared with the control group (Table 5). The hematology analysis and serum biochemistry analysis are summarized in Table 6 and Table 7. Some of the parameters showed statistical differences between the antrocin-treated and control groups. However, these data were within the normal reference range in rats, indicating the changes were not related to the test substance. In the positive control group, liver-associated enzymes, such as alanine transaminase (ALT), triglyceride (TG), high density lipoprotein-cholesterol (HDL-C) in male and female rats, and cholesterol in male rats, were statistically (*p <* 0.05) higher than in control groups, which indicated the hepatoxicity of sorafenib. For the parameters of urinalysis, such as volume, pH, specific gravity, glucose, protein, and urinary segments, etc., there were no significant differences in any group. In terms of organ weight change (Table 8), the liver weights in the female antrocin-treated groups and the kidney weights in the female 7.5 mg/kg antrocin group were significantly increased (*p <* 0.05) compared to the control group. Heart, liver, kidneys, spleen, and testes weights were significantly decreased (*p* 0.05) in male positive control group. The liver weight in female positive control group was significantly increased (*p <* 0.05) compared to control group. However, no specific gross lesions were found.

In the histopathological examination, no significant treatment-related lesions were found in the high dose of antrocin group (Tables S1 and S2). Only non-specific microscopic changes including mononuclear cell infiltration in the heart, prostate gland, and Harderian gland, cysts and infarct in the kidneys, and fatty change in the liver were observed in some of the control and antrocin-treated rats. However, there were no signs of toxicity attributed to antrocin since the lesions were viewed as spontaneous abnormalities that occur in SD rats, according to the incidence and severity score. In the positive control group, sorafenib 7.5 mg/kg, diffuse epiphysis hypertrophy in male and female rats, diffuse hypocellularity in the bone marrow, and diffuse atrophy in spleen in male rats, and an increase in multifocal well-developed follicles in the ovary in female rats were found.

### 3.2. Anti-Oxidant Activity

The total phenolic content, ferrous equivalent, and Trolox equivalents of 10 mg/mL antrocin were 0.016 ± 0.000 mM Catechin equivalents, 0.218 ± 0.004 mM Fe^2+^ equivalents, and 0.508 ± 0.003 mM Trolox equivalents, respectively. The data for antioxidant capacity is summarized in Table 9.

### 3.3. Antimutagenicity Activity

The results of the non-metabolized group (without S9 mixture) showed that the inhibition of TA98 strain mutation induced by 4-NQO with three nontoxic concentrations of 0.0125, 0.025, and 0.05 mg/plate. For the negative control group, results were 20.3 ± 5.7, and in the positive control group (4-NQO, 2.5 μg/plate), the number of reverse mutants was 117.0 ± 7.2, which was about 5 times more than the negative control group. The numbers of bacterial reverse mutants with 0.0125, 0.025, and 0.05 mg/plate antrocin were 83.0 ± 3.6, 67.7 ± 2.5, and 21.7 ± 1.5, respectively, and the inhibition was 35.17%, 51.03%, and 98.62%, respectively. The metabolism group (with S9 mixture) showed that antrocin inhibited the mutagenesis of TA98 induced by 2-AA. The negative control group was 22.3 ± 4.2, and the bacterial reverse mutants of the positive control group (2-AA, 25 μg/plate) was 6055.0 ± 661.0, which was about 275 times more than the negative control group. The numbers of bacterial reverse mutants of the 0.0125, 0.025, and 0.05 mg/plate antrocin were 5680.3 ± 640.7, 4959.0 ± 739.1, and 4024.0 ± 251.1, respectively, and the inhibition rates were 6.21%, 18.17%, and 33.67%, respectively. The experimental results show that antrocin has a significant inhibition effect on the direct mutagen 4-NQO, which is a strong anti-mutagenic agent. It has a good inhibitory effect and is a medium-strength antimutagenic agent. The results of antimutagenicity test in TA98 are summarized in Table 10.

The results of the non-metabolized group (without S9 mixture) showed that the inhibition of TA1535 strain mutation induced by sodium azide with three nontoxic concentrations of 0.0125, 0.025, and 0.05 mg/plate. The negative control group was 10.7 ± 2.5, and the positive control group (sodium azide, 5 μg/plate) was 1313.0 ± 42.0. The number of reverse mutants was 1313.0 ± 42.0, which was about 122 times more than that of the negative control group. The numbers of bacterial reverse mutants of 0.0125, 0.025, and 0.05 mg/plate were 1255.7 ± 47.7, 1095.0 ± 28.0, and 977.0 ± 18.1, respectively, and the inhibition was 4.40%, 16.74%, and 25.80%, respectively. The metabolism group (with S9 mixture) showed that antrocin inhibited the mutagenesis of TA1535 induced by 2-AA. The negative control group was 11.7 ± 2.1, and the bacterial reverse mutants of the positive control group (2-AA, 25 μg/plate) was 270.3 ± 7.5, which was about 23 times more than the negative control group. The numbers of bacterial reverse mutants of the 0.0125, 0.025, and 0.05 mg/plate antrocin were 248.0 ± 9.5, 217.3 ± 10.2, and 184.3 ± 3.8, respectively, and the inhibition rates were 8.63%, 20.49%, and 33.25%, respectively. The experimental results show that antrocin in TA1535 has a good inhibition effect on the direct mutagen sodium azide, which is a moderately strong antimutagenic agent. It is necessary to add S9 mixture to activate indirect mutagenesis. The indirect mutagen 2-AA had a good inhibitory effect and was a moderately strong antimutagenic agent. The results of the antimutagenicity test in TA1535 are summarized in Table 10.

## 4. Discussion

Research over the past few years has shown that genetic mutations are a key factor in cancer. Mutations can lead to malignant mutations in cells and destroy other cellular components. In this study, the total phenolic content and antioxidant capacity of antrocin were determined. In the antimutagenic assay of *Salmonella typhimurium* frame-shift mutation strain TA98, in the group without S9 mixture, 0.05 mg/plate antrocin had a very strong anti-mutagenic effect. In addition, with S9 metabolic activation, it still remains a moderately strong antimutagenic agent.

AC has been used as a traditional Chinese medicine for a long time, and it has proved to have several beneficial effects for human health, especially in cases of liver dysfunction. Not only AC but also its compounds are believed to have more effective medical utilization. Different kinds of AC products are currently commercially available, and the demand for AC is increasing. Subsequently, its safety and usage raise concerns. Several reports support the safety of AC for human use. Lin et al. investigated the safety of AC powder, which contains 94% mycelium and 5% fruiting bodies extract [6]. The Ames test, mammalian chromosomal aberration test, and micronucleus test were conducted following OECD guidelines. The results showed no potential genotoxic effects associated with the test article. In another AC toxicity report by Lo et al., solid-state-cultivated mycelial powder of AC showed no mutagenicity in Ames tests or micronucleus tests [24].

Despite the safety reports conducted for AC, the toxicity of antrocin remains unknown. In preliminary tests, antrocin showed no bacterial mutagenicity in *Salmonella typhimurium* TA98 or TA100 at the dose of 100 μg/plate. In regard to mammalian cells, antrocin showed cytotoxicity not only in different kinds of tumor cells at varying doses, but also in CHO-K1 cells. In the present study, the half maximal inhibitory concentration (IC_50_) of antrocin was about 122.7 μg/mL. However, the MTT method for detecting cytotoxicity is not the best endpoint suggested by the guideline, which may increase the occurrence of false positive results [25]. Regarding the absence of toxicity results in the short-term test of this experiment, test concentrations lower than the recommended cytotoxicity may lead to false negative results since there is no risk at low doses. Therefore, the toxicity of the test substance to the cells and the selection of the dose are very crucial. Nevertheless, considering the low doses used in the in vivo micronucleus test and the absence of myelotoxicity, it is possible that the exposure of bone marrow was not sufficient to induce a potential genotoxic effect. No potential genotoxic effects of antrocin were found in this study. Further genotoxicity tests need to be investigated.

In the 28-day oral toxicity test, several parameters presented statistically significant changes in hematology and biochemistry tests in the antrocin-treated group, but the data was still within the reference range for rats. Thus, the results were not dose-dependent, and no clinical or histopathological findings could support toxicity. Mononuclear cell infiltration in the heart, prostate gland, and Harderian gland was thought to be a background lesion, and may have been associated with diet, environment, and stress [26]. Tubular cysts and infarct of low severity and incidence were considered congenital lesions of rats [27]. Fatty change in the liver was associated with disruption in lipid metabolism, which could be caused by different agents [28]. In this study, we found only one female rat with slight macrovesicular fatty change, and it was viewed as an individual incidental finding. No treatment-related toxicity was observed in the study. However, due to limited amounts of available synthetic antrocin, further toxicity tests at higher dosages should be investigated.

In sorafenib-treated rats, multiorgan toxicity, including liver, bone, bone marrow, and ovaries, was observed in this 28-day oral toxicity study. Sorafenib is a tyrosine kinase inhibitor which is approved for a certain type of liver, kidney, and thyroid gland tumor. Sorafenib can interfere with several signal transduction pathways by inhibiting Raf serine/threonine kinases. It can also block vascular endothelial growth factor (VEGF) and platelet-derived growth factor receptor-β (PDGFR-β) to affect tumor angiogenesis [29]. In the drug report of the European Medicines Agency, Wistar rats were used for 28-day oral toxicity test, and they found abnormality when dosing above 25 mg/kg in clinical observation, hematology, biochemistry, and histopathology [30]. In our study, alanine transaminase (ALT), triglyceride (TG), high density lipoprotein-cholesterol (HDL-C), and cholesterol were significantly increased. Sorafenib is reported to have liver and pancreas toxicity, and the mechanism is assumed to the anti-angiogenesis effects which may lead to pancreas cells infarct and necrosis [31,32]. However, no histopathological evidence could prove pancreas toxicity in our study, suggesting that the dosage was too low to cause direct damage in liver and pancreas cells. Other lesions such as diffuse epiphysis hypertrophy in male and female rats, diffuse hypocellularity in the bone marrow, diffuse atrophy in spleen, and multifocal well-developed follicles increase were suggested to correlate to anti-angiogenesis effects [33]. Similar lesions were observed in other anti-angiogenesis drugs [34].

Sesquiterpene lactones (SLs) are a group of secondary metabolites that can be found in different plants, mostly derived from Asteraceae. In plants, SLs can provide good defense against bacterial, viral, and fungal infection [35]. Current research has found that it is also beneficial to human health, and SLs-rich plants have been used in folk medicine for a long time. SLs can act as anti-inflammatories and prevent tumorigenesis, and these effects are attributed to its unique structure [36]. Some of the SLs compounds have already gone through clinical trials, and have shown inspiring properties for targeting tumor cells [37].

Interestingly, we found lymphocytes slightly increased in antrocin-treated rats. In a previous study, some of the sesquiterpene lactones could enhance immune responses and ease the progression of tumor cells [38]. Since antrocin was reported to have anti-tumor effects, it is assumed that it affects immune responses as well.

## 5. Conclusions

In summary, we present anti-oxidant, three genotoxicity assays and a 28-day oral toxicity test in rats of a novel compound, antrocin. Base on the results of this study, antrocin has anti-oxidant activity, low potential for mutagenic and genotoxic effects, and the current highest dosage of 37.5 mg/kg/day of antrocin presented no adverse effect on the 28-day oral toxicity test in rats.

## Figures and Tables

**Table 1:** Crystal data and structure refinement of antrocin.

Empirical formula	C_15_ H_22_O_2_	
Formula weight	234.32	
Temperature	200 (2) K	
Wavelength	1.54178 Å	
Crystal system	Monoclinic	
Space group	P2_1_	
Unit cell dimensions	a = 6.1732 (2) Å	α = 90°
	b = 15.9936 (4) Å	β = 114.0949(6)°
	c = 7.1673 (2) Å	γ = 90°
Volume	645.98 (3) Å^3^	
Z	2	
Density (calculated)	1.205 Mg/m^3^	
Absorption coefficient	0.610 mm^−1^	
F(000)	256	
Crystal size	0.185 × 0.167 × 0.122 mm^3^	
Theta range for data collection	8.756 to 74.972°.	
Index ranges	−7 ≤ h ≤ 6, −20 ≤ k ≤ 19, −8 ≤ l ≤ 8	
Reflections collected	4464	
Independent reflections	2615 [R(int) = 0.0209]	
Completeness to theta = 67.679°	98.9%	
Absorption correction	Semi-empirical from equivalents	
Max. and min. transmission	0.7539 and 0.6924	
Refinement method	Full-matrix least-squares on F^2^	
Data/restraints/parameters	2615/1/156	
Goodness-of-fit on F^2^	1.049	
Final R indices [I > 2sigma(I)]	R1 = 0.0308, wR2 = 0.0834	
R indices (all data)	R1 = 0.0310, wR2 = 0.0836	
Absolute structure parameter	0.03(5)	
Extinction coefficient	n/a	
Largest diff. peak and hole	0.216 and −0.120 e.Å^−3^	

**Table 2:** Revertant changes of antrocin in *Salmonella* TAs mutagenicity test.

Group	Number of Revertant (Colony/Plate)				
	TA98	TA100	TA1535	TA102	TA1537
Without S9 metabolic activation					
Negative ^1^	271.0 ± 7.8 ^3^	168.3 ± 10.5	8.0 ± 1.4	271.0 ± 7.8	9.7 ± 2.9
Positive ^2^	2004.3 ± 604.1 *	2223.0 ± 53.2 *	1514.3 ± 63.6 *	2004.3 ± 604.1 *	397.3 ± 20.7 *
Antrocin					
0.003125	245.7 ± 10.4	170.7 ± 1.2	11.7 ± 2.4	245.7 ± 10.4	14.3 ± 2.6
0.00625	246.0 ± 16.4	169.3 ± 8.2	14.7 ± 4.0	246.0 ± 16.4	8.7 ± 0.5
0.0125	279.3 ± 4.5	166.7 ± 3.8	13.0 ± 2.9	279.3 ± 4.5	8.0 ± 0.8
0.025	278.3 ± 4.5	181.7 ± 5.7	10.7 ± 1.2	278.3 ± 4.5	13.0 ± 2.2
0.05	270.0 ± 11.2	166.7 ± 4.5	12.7 ± 2.9	270.0 ± 11.2	9.7 ± 0.9
With S9 metabolic activation					
Negative	35.3 ± 2.5	194.0 ± 5.9	16.3 ± 2.6	339.7 ± 9.6	9.3 ± 2.4
Positive	1881.3 ± 434.2 *	2099.7 ± 145.5 *	535.0 ± 138.6 *	1532.0 ± 204.2 *	269.0 ± 21.9 *
Antrocin					
0.003125	33.7 ± 1.2	194.3 ± 5.4	17.3 ± 0.5	352.0 ± 8.5	11.7 ± 1.9
0.00625	32.0 ± 1.6	185.0 ± 6.5	15.7 ± 6.1	326.7 ± 5.9	6.7 ± 2.4
0.0125	31.3 ± 3.4	189.0 ± 5.1	15.3 ± 3.3	327.3 ± 5.7	6.0 ± 0.8
0.025	34.3 ± 3.3	197.0 ± 8.8	18.0 ± 1.6	327.3 ± 7.7	10.3 ± 2.1
0.05	36.0 ± 4.3	195.3 ± 2.9	14.0 ± 2.4	322.0 ± 6.2	6.0 ± 1.4

^1^ Negative control for antrocin was added with ethanol. ^2^ Positive reagents without S9 reactions were 2.5 μg/plate 4-NQO for TA98, 5 μg/plate sodium azide for TA100; the positive reagent with S9 was 5 μg/plate 2-AA for all *Salmonella* strains. ^3^ Data are presented as mean ± SD (*n* = 3). * Significant difference of colonies with more than twice the negative control and treated groups at *p* < 0.05.

**Table 3:** Percentages of chromosomal aberration test after incubation with antrocin in CHO-K1 cells.

Group	Total Aberrations	Frequency of Chromosomal Aberration (%) ^1^
Without S9 (3 h)		
Negative control	17/300	5.7 ± 3.2 ^2^
Mitomycin C (2.5 μg/mL)	44/300	14.7 ± 2.3 *
Antrocin (μg/mL)		
25	13/300	4.3 ± 3.8
50	13/300	4.3 ± 2.9
100	16/300	5.3 ± 2.1
With S9 (3 h)		
Negative control	17/300	5.7 ± 0.6
Cyclophosphamide (25 μg/mL)	46/300	15.3 ± 2.3 *
Antrocin (μg/mL)		
25	16/300	5.3 ± 1.2
50	19/300	6.3 ± 2.1
100	19/300	6.3 ± 2.1
Without S9 (19 h)		
Negative control	28/300	9.3 ± 1.2
Mitomycin C (2.5 μg/mL)	86/300	32.0 ± 6.0 *
Antrocin (μg/mL)		
25	28/300	9.3 ± 1.2
50	31/300	10.3 ± 2.5
100	22/300	7.3 ± 1.5

^1^ Two slides were prepared and stained with Diff-Quik Kit for 3 steps and a total number of 300 metaphases were counted for each dosage. All results are expressed in number of aberrations per plate. ^2^ The number of cells with damaged chromosomes was recorded, from which the rate of mutation was calculated. Aberration rate (%) = (number of cells with damaged chromosomes/100) × 100. Data are expressed as mean ± SD, n = 3. * Significant difference between the negative control and treated groups at *p <* 0.05.

**Table 4:** Changes in reticulocytes and reticulocytes with micronuclei in the peripheral blood of male mice after gavage treatment with antrocin.

Group/	Dose (mg/kg)	RETs/1000RBCs (‰)	Mn-RETs/1000RETs (‰)
Intervals			
Male			
48 h			
NC ^1^	0	19.1 ± 5.2 ^2^	2.6 ± 0.8
PC	60	3.6 ± 2.1 *	22.9 ± 12.2 *
Antrocin	100	20.4 ± 2.4	2.2 ± 0.9
	125	20.8 ± 2.5	1.8 ± 0.8
	250	18.8 ± 1.9	1.5 ± 0.2
72 h			
NC	0	23.7 ± 5.2	2.2 ± 0.3
PC	60	3.9 ± 2.1 *	6.8 ± 3.7 *
Antrocin	100	31.0 ± 6.1	3.0 ± 1.0
	125	28.9 ± 5.0	2.1 ± 0.5
	250	26.5 ± 4.6	2.2 ± 0.5

^1^ NC: negative control; RETs: reticulocytes; RBCs: erythrocytes; Mn-RETs: micronucleated reticulocytes; PC: positive control (Cyclophosphamide 60 mg/kg bw, ip.). ^2^ Data are expressed as the mean ± SD (n = 5). * Significant difference compared with the negative control and treated groups at *p <* 0.05.

**Table 5:** Body weight and body weight gain changes for rats treated with antrocin at 0, 7, 14, 21 and 28 days in the 28-day oral toxicity study.

Sex/Group	Body Weight/Body Weight Gain (g)				
	0-Day	7-Day ^1^	14-Day ^2^	21-Day ^3^	28-Day ^4^
Male					
NC ^5^	181.8 ± 3.2 ^6^	240.8 ± 8.3	295.4 ± 22.0	341.1 ± 35.0	379.8 ± 45.5
		59.1 ± 8.7	54.5 ± 14.5	45.7 ± 13.7	38.7 ± 11.0
Sorafenib					
7.5 mg/kg	183.0 ± 9.8	221.3 ± 11.8	257.5 ± 14.5	282.1 ± 21.7	295.2 ± 43.3
		38.2 ± 3.7 *	36.2 ± 7.4 *	24.6 ± 11.7 *	13.1 ± 27.7
Antrocin					
7.5 mg/kg	181.4 ± 5.1	237.9 ± 13.6	295.0 ± 22.5	349.5 ± 34.3	389.6 ± 46.6
		56.5 ± 10.1	57.1 ± 8.9	54.5 ± 12.4	40.1 ± 12.5
37.5 mg/kg	183.9 ± 9.0	235.6 ± 9.6	289.0 ± 17.3	332.3 ± 21.0	367.6 ± 30.6
		51.8 ± 4.7	53.4 ± 13.9	43.2 ± 7.7	35.3 ± 12.9
Female					
NC	163.7 ± 6.0	187.1 ± 8.7	205.0 ± 7.1	223.4 ± 11.0	237.9 ± 16.5
		23.5 ± 5.7	17.8 ± 4.1	18.4 ± 4.0	14.5 ± 6.0
PC					
7.5 mg/kg	163.7 ± 7.8	184.7 ± 10.4	205.6 ± 12.5	217.9 ± 16.0	227.3 ± 21.1
		21.0 ± 5.6	20.9 ± 4.1	12.3 ± 5.1	9.4 ± 5.9
Antrocin					
7.5 mg/kg	163.3 ± 6.3	190.0 ± 9.0	212.9 ± 12.1	229.3 ± 14.6	246.6 ± 21.7
		26.7 ± 5.6	22.8 ± 6.0	16.4 ± 5.2	17.3 ± 7.9
37.5 mg/kg	162.4 ± 6.3	188.5 ± 7.9	212.0 ± 7.0	230.1 ± 9.7	241.7 ± 13.6
		26.1 ± 4.1	23.4 ± 3.6	18.1 ± 5.0	11.6 ± 6.3

^1^ Mean body weight (BW) gain on the 7th day (g) = (7th − 0th)BW (g). ^2^ Mean body weight gain on the 14th day (g) = (14th − 7th)BW (g). ^3^ Mean body weight gain on the 21st day (g) = (21st − 14th)BW (g). ^4^ Mean body weight gain on the 28th day (g) = (28th − 21st)BW (g). ^5^ NC: negative control (olive oil). ^6^ Data are expressed as the mean ± SD (n = 5). * Significant difference between the control and treated groups at *p* < 0.05.

**Table 6:** Hematological parameter changes of rats treated with antrocin in the 28-day oral toxicity study.

Group		NC ^2^	Sorafenib 7.5 mg/kg	Antrocin 7.5 mg/kg	Antrocin 37.5 mg/kg	NC ^2^	Sorafenib 7.5 mg/kg	Antrocin 7.5 mg/kg	Antrocin 37.5 mg/kg
Sex		Male				Female			
RBC ^1^	(10^6^/µL)	7.4 ± 0.3 ^3^	8.4 ± 0.5 *	7.7 ± 0.1	8.1 ± 0.6	7.9 ± 0.5 ^3^	7.7 ± 0.4	7.5 ± 0.6	8.0 ± 0.4
HGB	(g/dL)	14.7 ± 0.8	16.4 ± 1.0 *	15.1 ± 0.4	15.7 ± 0.9	15.0 ± 0.8	15.5 ± 0.6	14.7 ± 1.2	15.8 ± 0.5
HCT	(%)	44.6 ± 2.3	49.8 ± 2.3 *	45.8 ± 1.2	48.3 ± 3.2	46.6 ± 2.6	47.7 ± 1.7	45.0 ± 3.7	48.4 ± 2.0
MCV	(fL)	59.8 ± 1.0	59.2 ± 0.8	59.8 ± 1.5	59.8 ± 1.3	58.7 ± 1.1	61.8 ± 1.2 *	59.7 ± 1.7	60.6 ± 1.0 *
MCH	(pg)	19.8 ± 0.4	19.5 ± 0.3	19.7 ± 0.5	19.4 ± 0.6	18.9 ± 0.3	20.0 ± 0.5 *	19.5 ± 0.8	19.8 ± 0.5 *
MCHC	(g/dL)	33.1 ± 0.5	33.0 ± 0.7	32.9 ± 0.3	32.4 ± 0.6	32.2 ± 0.6	32.4 ± 0.8	32.7 ± 0.3	32.7 ± 0.4
PLT	(10^3^/µL)	940.6 ± 272.7	874.0 ± 307.7	1235.2 ± 20.9 *	1372.2 ± 161.8 *	1166.8 ± 128.0	754.2 ± 238.6 *	606.0 ± 430.1 *	1065.6 ± 199.8
WBC	(10^3^/µL)	4.0 ± 2.2 ^3^	6.0 ± 2.2	5.3 ± 2.0	6.4 ± 1.1	7.5 ± 4.5	7.1 ± 1.1	8.7 ± 3.4	7.8 ± 4.5
NEUT	(%)	22.3 ± 4.0	35.4 ± 12.0	18.9 ± 3.1	13.6 ± 3.7 *	12.9 ± 4.4	17.8 ± 4.6	9.6 ± 3.3	10.9 ± 3.4
LYMPH	(%)	70.1 ± 2.8	59.0 ± 10.7	77.2 ± 1.7 *	83.9 ± 3.9 *	83.0 ± 4.9	77.2 ± 5.7	86.1 ± 5.4	84.5 ± 4.1
MONO	(%)	2.9 ± 2.0	0.9 ± 0.5	2.7 ± 2.4	1.8 ± 1.1	2.9 ± 2.1	3.1 ± 1.7	2.3 ± 1.1	3.2 ± 2.0
EOSIN	(%)	4.6 ± 4.8	0.1 ± 0.1	1.2 ± 0.3	0.6 ± 0.4	1.1 ± 0.3	1.8 ± 1.6	2.1 ± 1.7	1.2 ± 0.3
BASO	(%)	0.1 ± 0.1	0.1 ± 0.1	0.0 ± 0.1	0.1 ± 0.1	0.1 ± 0.1	0.1 ± 0.1	0.0 ± 0.1	0.2 ± 0.2

^1^ RBC: red blood cell; HGB: hemoglobin; HCT: hematocrit; MCV: mean corpuscular volume; MCH: mean corpuscular hemoglobin; MCHC: mean corpuscular hemoglobin concentration; WBC: white blood cell.; LYMPH: lymphocyte; NEUT: neutrophil; MONO: monocyte; EOSIN: eosinophil; BASO: basophil. ^2^ NC: negative control (olive oil). ^3^ Data are expressed as the mean ± SD (n = 5). * Significant difference between the control and treated groups at *p* < 0.05.

**Table 7:** Serum biochemistry changes of rats in the 28-day oral toxicity study.

Group		NC ^2^	Sorafenib 7.5 mg/kg	Antrocin 7.5 mg/kg	Antrocin 37.5 mg/kg	NC ^2^	Sorafenib 7.5 mg/kg	Antrocin 7.5 mg/kg	Antrocin 37.5 mg/kg
Sex		Male				Female			
AST ^1^	(U/L)	61.0 ± 9.6 ^3^	106.2 ± 2.4 *	61.4 ± 4.3	60.2 ± 3.3	74.8 ± 6.1 ^3^	94.8 ± 6.4 *	58.0 ± 8.5 *	72.6 ± 7.4
ALT	(U/L)	27.4 ± 4.6	84.8 ± 13.7 *	28.2 ± 3.1	27.6 ± 2.1	31.0 ± 8.3	69.0 ± 7.6 *	24.2 ± 1.9	32.6 ± 6.2
ALP	(U/L)	257.2 ± 39.2	261.0 ± 148.9	238.4 ± 43.9	256.8 ± 44.0	128.2 ± 25.9	147.6 ± 53.2	147.4 ± 27.6	116.4 ± 35.7
CK	(U/L)	173.2 ± 37.7	229.2 ± 49.7	185.6 ± 96.1	156.4 ± 31.1	109.0 ± 15.2	137.2 ± 16.7 *	100.2 ± 30.8	129.8 ± 59.5
BUN	(mg/dL)	11.8 ± 1.3	14.8 ± 1.8 *	11.2 ± 1.3	11.6 ± 1.8	12.4 ± 2.2	14.0 ± 1.2	12.0 ± 1.0	11.0 ± 0.7
Creatinine	(mg/dL)	0.4 ± 0.0	0.4 ± 0.0	0.3 ± 0.0	0.3 ± 0.0	0.4 ± 0.1	0.4 ± 0.1	0.4 ± 0.0	0.3 ± 0.1
Cholesterol	(mg/dL)	43.4 ± 9.8 ^3^	100.8 ± 13.9 *	41.2 ± 6.3	44.0 ± 2.5	41.2 ± 8.6	76.2 ± 7.0 *	51.2 ± 9.8	47.0 ± 11.8
Amylase	(U/L)	1994.2 ± 191.5	1432.0 ± 348.8 *	1931.0 ± 241.1	2016.4 ± 289.7	1311.8 ± 143.3	1076.4 ± 197.5	1280.6 ± 111.3	1240.4 ± 239.9
Glucose	(mg/dL)	212.4 ± 37.6	176.6 ± 19.4	178.0 ± 25.7	178.0 ± 14.5	179.8 ± 19.9	143.0 ± 19.1 *	182.4 ± 42.9	138.2 ± 21.7 *
GGT	(U/L)	<1	<1	<1	<1	1.6	<1	1–2	1–2
LDH	(U/L)	164.2 ± 24.6	229.6 ± 108.3	219.6 ± 139.5	109.8 ± 33.5 *	82.4 ± 23.4	81.8 ± 23.8	92.4 ± 73.8	125.4 ± 96.2
TB	(mg/dL)	<0.1	<0.1	<0.1	<0.1	<0.1	<0.1	<0.1	<0.1
UA	(mg/dL)	2.0 ± 0.6	1.6 ± 0.4	1.5 ± 0.1	1.6 ± 0.3	1.5 ± 0.9	<0.9	0.9 ± 0.1	1.3 ± 0.6
Globulin	(g/dL)	1.9 ± 0.1 ^3^	1.6 ± 0.3	1.9 ± 0.1	2.0 ± 0.2	2.1 ± 0.3	2.0 ± 0.2	1.9 ± 0.1	2.2 ± 0.1
Albumin	(g/dL)	3.5 ± 0.1	3.1 ± 0.4	3.4 ± 0.1	3.5 ± 0.2	3.7 ± 0.2	3.5 ± 0.3	3.7 ± 0.2	3.8 ± 0.2
A/G		1.8 ± 0.1	1.9 ± 0.3	1.8 ± 0.1	1.8 ± 0.1	1.8 ± 0.1	1.8 ± 0.1	1.9 ± 0.2	1.7 ± 0.1
HDL-C	(mg/dL)	12.8 ± 1.3	25.2 ± 1.5 *	12.8 ± 1.5	15.2 ± 2.8	12.8 ± 3.3	23.2 ± 1.9 *	16.0 ± 3.4	14.6 ± 3.8
TP	(mg/dL)	5.4 ± 0.1	4.7 ± 0.7	5.3 ± 0.2	5.5 ± 0.4	5.8 ± 0.5	5.5 ± 0.4	5.6 ± 0.2	6.0 ± 0.3
TG	(mg/dL)	32.0 ± 7.1	181.4 ± 202.0	33.0 ± 8.1	46.2 ± 8.3 *	27.2 ± 2.9	59.6 ± 23.4 *	32.8 ± 5.2	27.2 ± 8.1
Ca^2+^	(mg/dL)	9.8 ± 0.4 ^2^	8.9 ± 0.4 *	9.7 ± 0.2	9.9 ± 0.4	10.0 ± 0.3	9.4 ± 0.6	9.9 ± 0.1	10.2 ± 0.2
Cl^−^	(mEq/dL)	105.0 ± 4.5	103.6 ± 2.3	106.4 ± 1.3	104.6 ± 2.1	103.8 ± 1.5	103.2 ± 1.6	105.6 ± 0.5 *	104.2 ± 1.3
K^+^	(mEq/dL)	7.6 ± 1.0	7.6 ± 0.7	6.5 ± 0.3 *	6.4 ± 0.6	5.2 ± 0.4	5.1 ± 0.1	5.2 ± 0.2	5.0 ± 0.7
Mg^2+^	(mg/L)	2.1 ± 0.3	2.2 ± 0.2	2.0 ± 0.1	2.1 ± 0.2	2.4 ± 0.2	2.3 ± 0.2	2.1 ± 0.1 *	2.4 ± 0.3
Na^+^	(mEq/dL)	140.6 ± 1.1	138.6 ± 0.9 *	141.8 ± 0.8	141.4 ± 2.3	141.2 ± 1.1	140.4 ± 2.1	139.2 ± 1.6	142.6 ± 1.1
Phosphate	(mg/dL)	8.7 ± 1.1	7.1 ± 1.5	9.1 ± 0.9	9.6 ± 1.6	9.3 ± 0.7	7.3 ± 1.1 *	8.5 ± 1.0	8.5 ± 0.5

^1^ AST: aspartate aminotransferase; ALT: alanine aminotransferase; ALP: alkaline phosphatase; BUN: blood urea nitrogen; CK: creatine kinase; LDH: lactate dehydrogenase; TB: total bilirubin; GGT: gamma glutamyl-transferase; UA: uric acid; A/G: albumin/globulin ratio; HDL-C: high density lipids-cholesterol; TG: triglyceride; TP: total protein. ^2^ NC: negative control (olive oil); ^3^ Data are expressed as the mean ± SD (n = 5); * Significant difference between the control and treated groups at *p* < 0.05.

**Table 8:** Absolute organ weight changes of rats in the 28-day oral toxicity study.

Sex/Group	Brain (g)	Heart (g)	Thymus (g)	Liver (g)	Kidney (g)	Adrenal (g)	Spleen (g)	Testis/Ovary (g)
Male								
NC ^1^	2.1 ± 0.1 ^2^	1.3 ± 0.1	0.5 ± 0.1	12.1 ± 2.2	3.0 ± 0.5	0.05 ± 0.01	0.6 ± 0.1	3.2 ± 0.4
Sorafenib 7.5 mg/kg	2.0 ± 0.1	0.9 ± 0.2 *	0.4 ± 0.3	7.9 ± 1.4 *	2.1 ± 0.4 *	0.05 ± 0.01	0.4 ± 0.1 *	2.7 ± 0.2 *
Antrocin								
7.5 mg/kg	2.1 ± 0.2	1.3 ± 0.2	0.6 ± 0.1	12.2 ± 1.9	2.8 ± 0.4	0.05 ± 0.01	0.6 ± 0.1	3.1 ± 0.2
37.5 mg/kg	2.0 ± 0.1	1.3 ± 0.2	0.4 ± 0.1	11.1 ± 1.2	2.7 ± 0.2	0.06 ± 0.00	0.6 ± 0.1	3.2 ± 0.2
Female								
NC	1.9 ± 0.2	0.8 ± 0.1	0.4 ± 0.1	6.8 ± 0.4	1.6 ± 0.1	0.06 ± 0.01	0.5 ± 0.1	0.10 ± 0.02
Sorafenib 7.5 mg/kg	2.0 ± 0.1	0.7 ± 0.0	0.5 ± 0.1	7.3 ± 0.1*	1.7 ± 0.1	0.06 ± 0.01	0.4 ± 0.0	0.09 ± 0.01
Antrocin								
7.5 mg/kg	1.9 ± 0.1	0.9 ± 0.1	0.4 ± 0.1	8.5 ± 0.7*	1.8 ± 0.1*	0.05 ± 0.01	0.4 ± 0.1	0.07 ± 0.02
37.5 mg/kg	2.0 ± 0.1	1.0 ± 0.1	0.4 ± 0.1	7.8 ± 0.5*	1.7 ± 0.1	0.05 ± 0.01	0.4 ± 0.0	0.10 ± 0.01

^1^ NC: negative control (olive oil). ^2^ Data are expressed as the mean ± SD (n = 5). * Significant difference between the control and treated groups at *p* < 0.05.

**Table 9:** The antioxidant properties of 10 mg/mL antrocin.

Components	Total Phenolic Contents (mM Catechin Equivalents)	Ferric Reducing Ability of Plasma (mM Ferrous Equivalent)	Trolox Equivalent Antioxidant Capacity (mM Trolox Equivalents)
Antrocin	0.016 ± 0.000 ^1^	0.218 ± 0.004	0.508 ± 0.003

^1^ Data are expressed as mean ± SD, n = 3.

**Table 10:** Revertant changes of antrocin in *Salmonella* TA98 and TA1535 antimutagenicity test.

Group	Number of Revertant (Colony/Plate)	
	TA98	TA1535
Without S9 metabolic activation		
Negative ^1^	20.3 ± 5.7 ^3^	10.7 ± 2.5
Positive ^2^	117.0 ± 7.2 *	1313.0 ± 42.0 *
Antrocin		
0.0125	83.0 ± 3.6 (35.17%)	1255.7 ± 47.7 (4.40%)
0.025	67.7 ± 2.5 (51.03%)	1095.0 ± 28.0 (16.74%)
0.05	21.7 ± 1.5 (98.62%)	977.0 ± 18.1 (25.80%)
With S9 metabolic activation		
Negative	22.3 ± 4.2	11.7 ± 2.1
Positive	6055.0 ± 661.0 *	270.3 ± 7.5 *
Antrocin		
0.0125	5680.3 ± 640.7 (6.21%)	248.0 ± 9.5 (8.63%)
0.025	4959.0 ± 739.1 (18.17%)	217.3 ± 10.2 (20.49%)
0.05	4024.0 ± 251.1 (33.67%)	184.3 ± 3.8 (33.25%)

^1^ Negative control for antrocin was added with ethanol. ^2^ Positive reagents without S-9 mix reactions were 2.5 μg/plate 4-nitroquinoline-N-oxide for TA98, 5 μg/plate sodium azide for TA1535; positive reagent with S-9 mix was 25 μg/plate 2-aminoanthracene for TA98 and TA1535. ^3^ Data are presented as mean ± SD (*n* = 3). * Significant difference of colonies more than two folds of negative control and treated groups at *p* < 0.05.

## Data Availability

Not applicable.

## Supplementary Materials

The following supporting information can be downloaded at: https://www.mdpi.com/article/10.3390/toxics11060547/s1, Table S1: Summary of pathological incidence of male rats in the side effect observation of the antrocin in the 28-day oral toxicity study; Table S2: Summary of pathological incidence of female rats in the side effect observation of the antrocin in the 28-day oral toxicity study.
