# Supplementary material for: Anti-Oxidant, Anti-Mutagenic Activity and Safety Evaluation of Antrocin
Source: Toxics. 2023 Jun 20;11(6):547. doi: 10.3390/toxics11060547 (PMC10301922; doi:10.3390/toxics11060547)
Supplement: Supplementary file 1 [file toxics-11-00547-s001.zip › toxics-2429045-supplementary.pdf]

Table S1. Summary of pathological incidence of male rats in the side effect observation of the antrocin in the 28-day oral toxicity study.

Organ	Histopathological lesions	Group/Male			
		NC[1]	PC	Antrocin 7.5 mg/kg	Antrocin 37.5 mg/kg
Adrenal gland					
	Congestion, cortex, multifocal, slight[2]	-	1/5[3]	-	-
Aorta		-	-	-	-
Brain					
Bone					
Femur					
	Hypertrophy, epiphysis, diffuse, slight to moderate/severe	0/5	5/5	0/5	0/5
	Score value	0	3.2±0.7[*, 4]	0	0
Bone marrow					
Femur					
	Hypocellularity, diffuse, slight to moderate/severe	0/5	3/5	0/5	0/5
	Score value	0	1.8±1.5[*]	0	0
Sternum					
	Hypocellularity, diffuse, moderate	0/5	3/5	0/5	0/5
	Score value	0	1.8±1.5[*]	0	0
Coagulative gland		-	-	-	-
Epididymis					
	Granuloma, spermatic, focal, moderate	-	1/5	-	-
Esophagus		-	-	-	-
Eyes		-	-	-	-
Harderian gland		-	-	-	-
Heart					
	Infiltration, mononuclear cell, focal, minimal to slight	1/5	1/5	1/5	1/5
Intestine, small					
Duodenum		-	-	-	-
Jejunum		-	-	-	-
Ileum		-	-	-	-
Intestine, large					
Caecum		-	-	-	-
Colon		-	-	-	-

Organ	Histopathological lesions	Group/Male			
		NC[1]	PC	Antrocin 7.5 mg/kg	Antrocin 37.5 mg/kg
Rectum		-	-	-	-
Kidney					
	Cast, tubule, focal, minimal	1/5	3/5	-	-
	Cyst, tubule, focal, slight	-	-	1/5	-
	Infarct, tubule, focal, slight	-	-	2/5	1/5
Liver		-	-	-	-
Lung		-	-	-	-
Lymph node		-	-		
Cervical		-	-	-	-
Mesenteric		-	-	-	-
Mammary gland		-	-	-	-
Optic nerve		-	-	-	-
Pancreas		-	-	-	-
Pituitary		-	-	-	-
Parathyroid gland		-	-	-	-
Prostate gland					
	Infiltration, mononuclear cell, focal, minimal to slight	1/5	-	-	2/5
Salivary gland					
Mandibular lobe		-	-	-	-
Sublingual lobe		-	-	-	-
Sciatic nerve		-	-	-	-
Seminal vesicle		-	-	-	-
Skeletal muscle		-	-	-	-
Skin		-	-	-	-
Spinal cord		-	-	-	-
Spleen	Atrophy, red pulp, diffuse, slight to moderate	0/5	5/5	0/5	0/5
	Score value	0	2.2±0.4*	0	0
Stomach		-	-	-	-
Testis		-	-	-	-
Thymus		-	-	-	-
Thyroid gland		-	-	-	-
Tongue		-	-	-	-
Trachea		-	-	-	-

Organ	Histopathological lesions	Group/Male			
		NC[1]	PC	Antrocin 7.5 mg/kg	Antrocin 37.5 mg/kg
Urinary bladder		-	-	-	-

[1] NC: negative control (olive oil); PC: positive control (sorafenib 7.5 mg/kg); -: No significant lesion. [2] Degree of lesions was graded from one to five depending on severity: 1 = minimal (< 1%); 2 = slight (1-25%); 3 = moderate (26-50%); 4 = moderate/severe (51-75%); 5 = severe/high (76-100%). [3] Incidence: Affected rats/ Total examined rats (n = 5). [4] The final numerical score was calculated by dividing the sum of the number per grade of affected rats by the total number of examined rats. [*] Significant difference between the control and treatment groups at $p<0.05$.

Table S2. Summary of pathological incidence of female rats in the side effect observation of the antrocin in the 28-day oral toxicity study.

Organ	Histopathological lesions	Group/Female			
		NC[1]	PC	Antrocin 7.5 mg/kg	Antrocin 37.5 mg/kg
Adrenal gland					
	Congestion, cortex, focal, slight[2]	-	1/5[3]	-	-
Aorta		-	-	-	-
Brain					
Bone					
Femur					
	Hypertrophy, epiphysis, diffuse, slight to moderate/severe	0/5	5/5	0/5	0/5
	Score value	0	3.2±0.4[*, 4]	0	0
Bone marrow					
Cervix		-	-	-	-
Esophagus		-	-	-	-
Eyes		-	-	-	-
Harderian gland					
	Infiltration, mononuclear cell, focal, minimal	-	-	-	1/5
Heart		-	-	-	-
Intestine, small					
Intestine, large					
Kidney					
	Cast, tubule, focal, slight	-	1/5	-	-
	Cyst, tubule, focal, slight	1/5	-	-	-
	Infarct, tubule, focal, slight	-	1/5	-	-
Liver					
	Fatty change, multifocal, slight	-	-	1/5	-
Lung		-	-	-	-
Lymph node					
Mammary gland		-	-		
Optic nerve		-	-	-	-
Ovary					
	Increase, follicle, multifocal, moderate to moderate/severe	0/5	4/4	0/5	0/5

Organ	Histopathological lesions	Group/Female			
		NC[1]	PC	Antrocin 7.5 mg/kg	Antrocin 37.5 mg/kg
	Total counted number of well-developed follicles	10.2±1.9	44.2±15.6*	12.6±6.1	10.8±4.8
Oviduct		-	-	-	-
Pancreas		-	-	-	-
Pituitary		-	-	-	-
Parathyroid gland		-	-	-	-
Salivary gland		-	-	-	-
Sciatic nerve		-	-	-	-
Skeletal muscle		-	-	-	-
Skin		-	-	-	-
Spinal cord		-	-	-	-
Spleen		-	-	-	-
Stomach		-	-	-	-
Thymus		-	-	-	-
Thyroid gland		-	-	-	-
Tongue		-	-	-	-
Trachea		-	-	-	-
Urinary bladder		-	-	-	-
Uterus		-	-	-	-
Vagina		-	-	-	-

[1] NC: negative control (olive oil); PC: positive control (sorafenib 7.5 mg/kg); -: No significant lesion.[2] Degree of lesions was graded from one to five depending on severity: 1 = minimal (< 1%); 2 = slight (1-25%); 3 = moderate (26-50%); 4 = moderate/severe (51-75%); 5 = severe/high (76-100%). [3] Incidence: Affected rats/ Total examined rats (n = 5). [4] The final numerical score was calculated by dividing the sum of the number per grade of affected rats by the total number of examined rats. * Significant difference between the control and treatment groups at $p<0.05$.
